# Synthesis and biological evaluation of 2-styrylquinolines as antitumour agents and EGFR kinase inhibitors: molecular docking study

**DOI:** 10.1080/14756366.2017.1407926

**Published:** 2017-12-18

**Authors:** Magda A.-A. El-Sayed, Walaa M. El-Husseiny, Naglaa I. Abdel-Aziz, Adel S. El-Azab, Hatem A. Abuelizz, Alaa A.-M. Abdel-Aziz

**Affiliations:** a Department of Pharmaceutical Organic Chemistry, Faculty of Pharmacy, Mansoura University, Mansoura, Egypt;; b Department of pharmaceutical chemistry, Faculty of pharmacy, Horus university, New Damietta, Egypt;; c Department of Medicinal Chemistry, Faculty of Pharmacy, Mansoura University, Mansoura, Egypt;; d Department of Pharmaceutical Chemistry, College of Pharmacy, King Saud University, Riyadh, Saudi Arabia;; e Department of Organic Chemistry, Faculty of Pharmacy, Al-Azahr University, Cairo, Egypt

**Keywords:** Styrylquinoline, thiadiazole, synthesis, antitumour, EGFR kinase inhibitors, molecular docking

## Abstract

A new series of 4,6-disubstituted 2-(4-(dimethylamino)styryl)quinoline **4a**,**b**–**9a**,**b** was synthesized by the reaction of 2-(4-(dimethylamino)styryl)-6-substituted quinoline-4-carboxylic acids **3a**,**b** with thiosemicarbazide, *p*-hydroxybenzaldehyde, ethylcyanoacetate, and 2,4-pentandione. In addition, the antitumour activity of all synthesized compounds **3a**,**b**–**9a**,**b** was studied via MTT assay against two cancer cell lines (HepG2 and HCT116). Furthermore, epidermal growth factor receptor (EGFR) inhibition, using the most potent antitumour compounds, **3a**, **3b**, **4a**, **4b**, and **8a**, was evaluated. The interpretation of the results showed clearly that the derivatives **3a**, **4a**, and **4b** exhibited the highest antitumour activities against the tested cell lines HepG2 and HCT116 with IC_50_ range of 7.7–14.2 µg/ml, in comparison with the reference drugs 5-fluorouracil (IC_50_ = 7.9 and 5.3 µg/ml, respectively) and afatinib (IC_50_ = 5.4 and 11.4 µg/ml, respectively). *In vitro* EGFR screening showed that compounds **3a**, **3b**, **4a**, **4b**, and **8a** exhibited moderate inhibition towards EGFR with IC_50_ values at micromolar levels (IC_50_ range of 16.01–1.11 µM) compared with the reference drugs sorafenib (IC_50_ = 1.14 µM) and erlotinib (IC_50_ = 0.1 µM). Molecular docking was performed to study the mode of interaction of compounds **3a** and **4b** with EGFR kinase.

## Graphical Abstract

**Figure:**
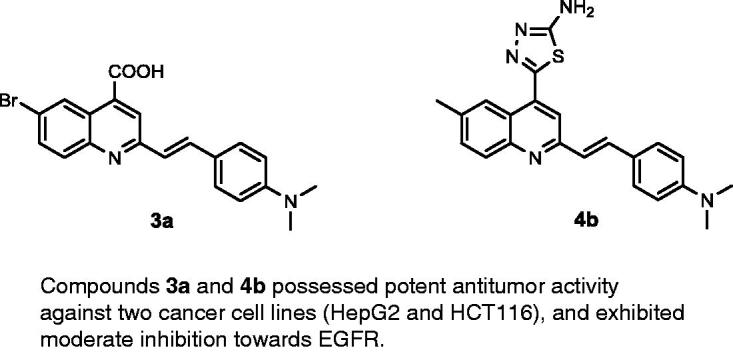

## 1. Introduction

**Figure 1.**
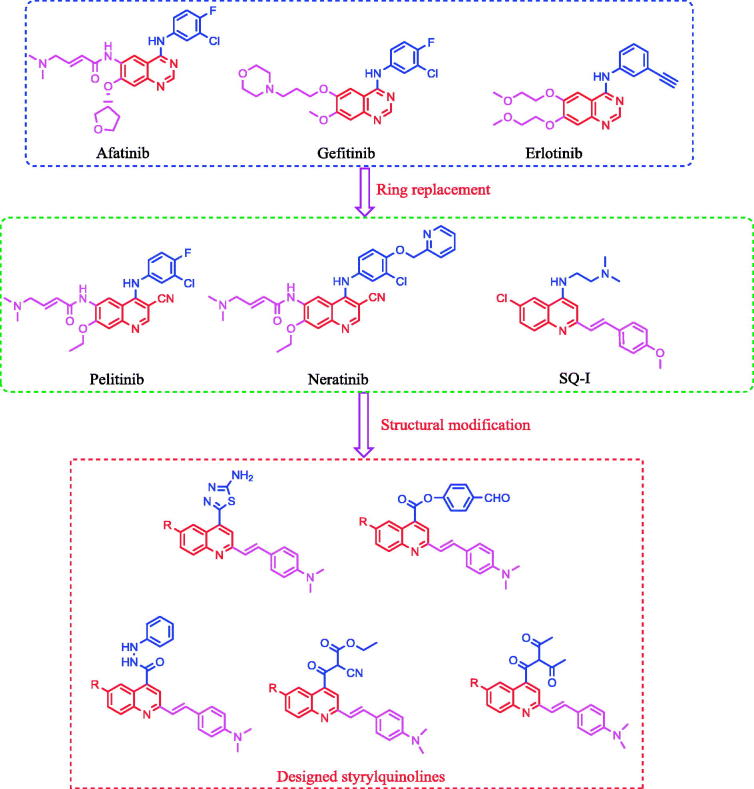
Reported EGFR inhibitors and antitumour agents, and design of the newly synthesized 2-styrylquinolines.

Development of a novel antitumour drug with potent activity remains critically important due to the majority of human deaths globally being attributable to cancer^1–13^. Epidermal growth factor receptors (EGFR) are an important class of kinase enzymes used in cancer treatment, which are overexpressed in several tumours, such as brain, liver, colon, prostate, breast, and non-small-cell lung cancers^14–18^. The inhibition of EGFR is affected by blocking tyrosine kinase at ATP-binding sites with small molecules, such as quinazoline derivatives^19–22^. Recently, afatinib, gefitinib, and erlotinib (Figure 1), quinazoline derivatives designed to inhibit EGFR kinase, have been approved by the FDA for the treatment of non-small cell lung and breast cancers^23–29^. Moreover, it was reported that the nitrogen atom at position-3 of the quinazoline core formed water-mediated hydrogen bond with Thr^766^ (gatekeeper residue) within the EGFR pocket^29^^,^^30^. On the contrary, bioisosteric replacement of the quinazoline ring system with quinoline, through conversion of the nitrogen atom at position-3 by carbon or carbonitrile fragments, yielded quinoline derivatives such as neratinib and pelitinib (Figure 1), which are potent EGFR kinase inhibitors^31–38^. This bioisosteric replacement^39^ did not require a water molecule to mediate the binding with the amino acid residue Thr^766^. 2-Styrylquinoline (SQ) derivatives have been reported as promising antitumour compounds against several tumour cell lines^40–43^. Recently, a series of 2-styryl-4-aminoquinoline (SQ-I, Figure 1) was developed which possessed potent *in vitro* antiproliferative activity against lung, colon, and liver cancer cell lines, comparable to gefitinib^44^.

The above-mentioned results encouraged us in designing and synthesizing a series of SQs, which are anticipated to be as potent as structurally related quinazoline bioisosteres. The antitumour activity of the target compounds was evaluated using two tumour cell lines, namely, human hepato-cellular carcinoma cell line (HepG2) and human colorectal carcinoma cell line (HCT116)^45–48^. In addition, some compounds were evaluated for their inhibitory activity against the EGFR tyrosine kinase enzyme. Moreover, a molecular docking method was used to study the putative binding mode of the target molecules into the receptor pocket of EGFR kinase^1^^,^^8–9^^,^^12^^,^^30^.

## 2. Experimental

### 2.1. Chemistry

Melting points (°C) were recorded using Stuart melting point apparatus and were uncorrected. IR spectra were recorded on a Mattson 5000 FT-IR spectrometer (in cm^−1^) (Mattson Instruments, Cambridge, UK) using KBr disk at the Faculty of Pharmacy, Mansoura University. ^1 ^H-NMR and ^13^C NMR spectra were recorded on a Bruker Avance spectrometer (400 MHz) in DMSO-d_6_ at Georgia State University, Atlanta, GA. The chemical shifts in ppm are expressed in δ units, using tetramethylsilane as an internal standard, and coupling constants in Hz. Mass spectrum analyses were performed on Thermo Fisher Scientific (Waltham, MA, USA) SID GC GC/MS, DSQ II in the Faculty of Science, Mansoura University. Reaction times were determined using a TLC technique on silica gel plates 60 F_245_E. Merck, and the spots were visualised by U.V. (366, 245 nm). Biological screening was conducted at the Pharmacognosy Department, Faculty of Pharmacy, Mansoura University. Compound **3a** was prepared according to its previous report^49^.

#### 2.1.1. Synthesis of compound 3b

A mixture of the appropriate 5-substituted isatin (5 mmol), 4-(4-(dimethylamino)phenyl)but-3-en-2-one (**1**) (0.945 g, 5 mmol) and potassium hydroxide (1.28 g, 23 mmol) in 50% aqueous ethanol (20 ml) was heated under reflux for 24 h. The reaction mixture was then diluted with 30% aqueous ethanol solution (20 ml) and neutralised with 50% acetic acid. The precipitated solid was filtered, dried, and crystallised from ethanol.

##### 2.1.1.1. 2–(4-(Dimethylamino)styryl)-6-methylquinoline-4-carboxylic acid (3b)

Light yellow crystals; yield: 78%; m.p. 275 °C. IR (KBr) γ/cm^−1^: 3400–3448 (br, OH), 1681 (C = O acid). ^1 ^H-NMR (400 MHz-DMSO-d_6_): δ 2.28 (s, 3 H, Ar-CH_3_), 3.00 (s, 6 H, 2CH_3_), 6.50 (d, *J* = 8 Hz, 1 H, styryl-H), 6.62 (d, *J* = 4 Hz, 1 H, styryl-H), 6.80 (d, 2 H, Ar-H), 7.45 (d, 2 H, Ar-H), 7.57 (d, *J* = 8 Hz, 1 H, Ar-H), 7.92 (s, 1 H, Ar-H), 8.03 (d, *J* = 8 Hz, 1 H, Ar-H), 8.35 (s, 1 H, Ar-H), 11.69 (s, 1 H, COOH, D_2_O exchangeable). ^13^C NMR (100 MHz-DMSO-d_6_): δ 21.5, 40.2, 111.1, 115.2, 124.5, 125.3, 126.8, 127.3, 127.5, 128.8, 129.9, 130.5, 132.1, 134.2, 144.9, 150.2, 166.8. MS (*m/z*%): 333 (M^+^ + 1, 10.55), 332 (M^+^, 42.60), 204 (100). Anal. Calcd for C_21_H_20_N_2_O_2_ (332.40): C, 75.88; H, 6.06; N, 8.43. Found: C, 75.91; H, 6.08; N, 8.47.
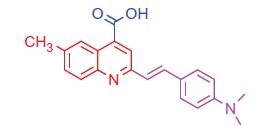


#### 2.1.2. General method for synthesis of compounds 4a,b

Phosphorus oxychloride (4.6 g, 34 mmol) was added dropwise to an ice-cold mixture of compounds **3a**,**b** and thiosemicarbazide (0.91 gm, 10 mmol) and the reaction mixture was heated under reflux for 8 h. The reaction mixture was cooled, poured into ice-cold water, and neutralised with 10% sodium carbonate solution. The precipitated solid was filtered, washed with water, dried, and crystallised from ethanol.

##### 2.1.2.1. 5-(6-Bromo-2–(4-(dimethylamino)styryl)quinolin-4-yl)-1,3,4-thiadiazol-2-amine (4a)

White crystals; yield: 82%; m.p. 223 °C. IR (KBr) γ/cm^−1^: 3380 (NH_2_), 685 (C-S-C). ^1 ^H-NMR (400 MHz-DMSO-d_6_): δ 3.20 (s, 6 H, 2CH_3_), 6.53 (d, *J* = 8 Hz, 1 H, styryl-H), 6.61 (d, *J* = 4 Hz, 1 H, styryl-H), 6.86 (d, 2 H, Ar-H), 7.53 (d, 2 H, Ar-H), 7.54 (d, *J* = 8 Hz, 1 H, Ar-H), 8.00 (s, 1 H, Ar-H), 8.13 (d, *J* = 8 Hz, 1 H, Ar-H), 8.29 (s, 1 H, Ar-H), 8.97 (s, 2 H, NH_2_, D_2_O exchangeable). MS (*m/z*%): 453 (M^+^ + 1, 35.14), 451 (M^+^ –1, 62.03), 324 (100). Anal. Calcd for C_21_H_18_BrN_5_S (452.37): C, 55.76; H, 4.01; N, 15.48. Found: C, 55.80; H, 4.06; N, 15.51.
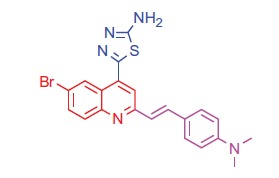


##### 2.1.2.2. 5-(2-(4-(Dimethylamino)styryl)-6-methylquinolin-4-yl)-1,3,4-thiadiazol-2-amine (4b)

White crystals; yield: 85%; m.p. 226 °C. IR (KBr) γ/cm^−1^: 3384 (NH_2_), 690 (C-S-C). ^1 ^H-NMR (400 MHz-DMSO-d_6_): δ 2.20 (s, 3 H, Ar-CH_3_), 3.14 (s, 6 H, 2CH_3_), 6.48 (d, *J* = 8 Hz, 1 H, styryl-H), 6.60 (d, *J* = 4 Hz, 1 H, styryl-H), 6.78 (d, 2 H, Ar-H), 7.45 (d, 2 H, Ar-H), 7.61 (d, *J* = 8 Hz, 1 H, Ar-H), 8.10 (s, 1 H, Ar-H), 8.17 (d, *J* = 8 Hz, 1 H, Ar-H), 8.33 (s, 1 H, Ar-H), 8.91 (s, 2 H, NH_2_, D_2_O exchangeable). ^13^C NMR (100 MHz-DMSO-d_6_): δ 19.8, 40.9, 110.5, 116.4, 121.2, 122.8, 126.3, 127.1, 128.2, 130.0, 131.2, 132.5, 133.3, 142.6, 143.4, 151.0, 157.1, 162.0, 173.7. MS (*m/z*%): 389 (M^+^ + 2, 8.85), 388 (M^+^ + 1, 42.21), 387 (M^+^, 53.20), 259 (100). Anal. Calcd for C_22_H_21_N_5_S (387.50): C, 68.19; H, 5.46; N, 18.07; S, 8.27. Found: C, 68.22; H, 5.50; N, 18.13; S, 8.29.
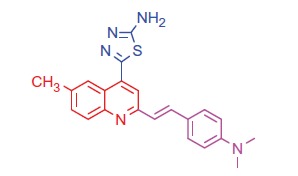


#### 2.1.3. General method for synthesis of 2-(4-(dimethylamino)styryl)-6-substituted quinoline-4-carbonyl chlorides (5a,b)

Thionyl chloride (4.17 g, 30 mmol) was added to quinolone carboxylic acid **3** (10 mmol). The reaction mixture was heated under reflux for 3 h. After cooling, it was evaporated under reduced pressure and the obtained solid was used directly in the next step without further purification.

#### 2.1.4. General method for synthesis of compounds 6a,b and 7a,b

A mixture of the acid chloride **5** (10 mmol), 4-hydroxybenzaldehyde (1.22 g, 10 mmol) or phenyl hydrazine (1.08 g, 10 mmol) and potassium carbonate (1.38 g, 10 mmol) in dimethylformamide (25 ml) was heated at 90 °C for 24 h. After cooling, the reaction mixture was poured into ice water. The obtained solid was filtered, washed with water, dried, and crystallised from ethanol to give pure products.

##### 2.1.4.1. 4-Formylphenyl 6-bromo-2-(4-(dimethylamino)styryl)quinoline-4-carboxylate (6a)

Buff crystals; yield: 90%; m.p. 242 °C. IR (KBr) γ/cm^−1^: 1715 (C = O aldehyde), 1690 (C = O ester). ^1 ^H-NMR (400 MHz-DMSO-d_6_): δ 2.96 (s, 6 H, 2CH_3_), 6.37 (d, *J* = 8 Hz, 1 H, styryl-H), 6.42 (d, *J* = 4 Hz, 1 H, styryl-H), 6.65 (d, 2 H, Ar-H), 6.82 (d, 2 H, Ar-H), 7.48 (d, *J* = 8 Hz, 1 H, Ar-H), 7.56 (d, 2 H, Ar-H), 7.61 (d, 2 H, Ar-H), 8.07 (s, 1 H, Ar-H), 8.13 (d, *J* = 8 Hz, 1 H, Ar-H), 8.35 (s, 1 H, Ar-H), 9.07 (s, 1 H, CHO). MS (*m/z*%): 502 (M^+^ + 1, 6.73), 501 (M^+^, 42.52), 500 (M^+^–1, 60.21), 373 (100). Anal. Calcd for C_27_H_21_BrN_2_O_3_ (501.38): C, 64.68; H, 4.22; N, 5.59. Found: C, 64.72; H, 4.26; N, 5.63.
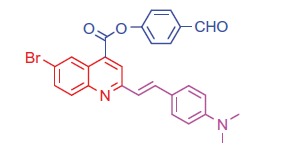


##### 2.1.4.2. 4-Formylphenyl 2-(4-(dimethylamino)styryl)-6-methylquinoline-4-carboxylate (6 b)

Pale brown crystals; yield: 92%; m.p. 248 °C. IR (KBr) γ/cm^−1^: 1710 (C = O aldehyde), 1695 (C = O ester). ^1 ^H-NMR (400 MHz-DMSO-d_6_): δ 2.30 (s, 3 H, Ar-CH_3_), 3.12 (s, 6 H, 2CH_3_), 6.39 (d, *J* = 8 Hz, 1 H, styryl-H), 6.40 (d, *J* = 4 Hz, 1 H, styryl-H), 6.85 (d, 2 H, Ar-H), 7.47 (d, *J* = 8 Hz, 1 H, Ar-H), 7.56 (d, 2 H, Ar-H), 7.62 (d, 2 H, Ar-H), 7.84 (d, 2 H, Ar-H), 8.11 (s, 1 H, Ar-H), 8.18 (d, *J* = 8 Hz, 1 H, Ar-H), 8.32 (s, 1 H, Ar-H), 9.13 (s, 1 H, CHO). MS (*m/z*%): 437 (M^+^ + 1, 22), 436 (M^+^, 37), 308 (100). Anal. Calcd for C_28_H_24_N_2_O_3_ (436.51): C, 77.04; H, 5.54; N, 6.42. Found: C, 77.08; H, 5.57; N, 6.46.
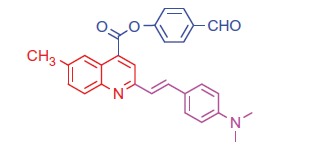


##### 2.1.4.3. 6-Bromo-2-(4-(dimethylamino)styryl)-N'-phenylquinoline-4-carbohydrazide (7a)

Brown crystals; yield: 89%; m.p. 232 °C. IR (KBr) γ/cm^−1^: 3362 (NH), 1650 (C = O amide). ^1 ^H-NMR (400 MHz-DMSO-d_6_): δ 3.12 (s, 6 H, 2CH_3_), 4.92 (s, 1 H, NH, D_2_O exchangeable), 5.20 (s, 1 H, NH, D_2_O exchangeable), 6.39 (d, *J* = 8 Hz, 1 H, styryl-H), 6.40 (d, *J* = 4 Hz, 1 H, styryl-H), 6.80 (d, 2 H, Ar-H), 7.47 (d, *J* = 8 Hz, 1 H, Ar-H), 7.56 (d, 2 H, Ar-H), 8.04 (s, 1 H, Ar-H), 8.18 (d, *J* = 8 Hz, 1 H, Ar-H), 8.21–8.29 (m, 3 H, Ar-H), 8.32 (s, 1 H, Ar-H), 8.41–8.48 (m, 2 H, Ar-H). ^13^C NMR (100 MHz-DMSO-d_6_): δ 42.1, 111.3, 114.0, 114.9, 118.5, 121.8, 123.7, 125.3, 126.9, 127.2, 129.1, 130.2, 131.7, 132.2, 138.9, 142.4, 143.8, 148.6, 150.0, 155.4, 163.9. MS (*m/z*%): 488 (M^+^ + 1, 18.52), 487 (M^+^, 56.12), 486 (M^+^–1, 68.03), 359 (100). Anal. Calcd for C_26_H_23_BrN_4_O (487.40): C, 64.07; H, 4.76; N, 11.50. Found: C, 64.11; H, 4.79; N, 11.54.
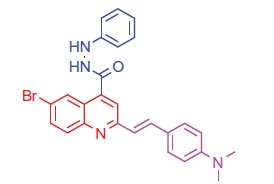


##### 2.1.4.4. 2-(4-(Dimethylamino)styryl)-6-methyl-N'-phenylquinoline-4-carbohydrazide (7 b)

Brown crystals; yield: 86%; m.p. 228 °C. IR (KBr) γ/cm^−1^: 3365 (NH), 1655 (C = O amide). ^1 ^H-NMR (400 MHz-DMSO-d_6_): δ 2.41 (s, 3 H, Ar-CH_3_), 3.33 (s, 6 H, 2CH_3_), 4.85 (s, 1 H, NH, D_2_O exchangeable), 5.21 (s, 1 H, NH, D_2_O exchangeable), 6.28 (d, *J* = 8 Hz, 1 H, styryl-H), 6.39 (d, *J* = 4 Hz, 1 H, styryl-H), 6.84 (d, 2 H, Ar-H), 7.55 (d, 2 H, Ar-H), 7.60 (d, *J* = 8 Hz, 1 H, Ar-H), 8.02 (s, 1 H, Ar-H), 8.10 (d, *J* = 8 Hz, 1 H, Ar-H), 8.19–8.26 (m, 3 H, Ar-H), 8.32 (s, 1 H, Ar-H), 8.39–8.47 (m, 2 H, Ar-H). MS (*m/z*%): 423 (M^+^ + 1, 32.31), 422 (M^+^, 55.17), 294 (100). Anal. Calcd for C_27_H_26_N_4_O (422.53): C, 76.75; H, 6.20; N, 13.26. Found: C, 76.79; H, 6.28; N, 13.30.
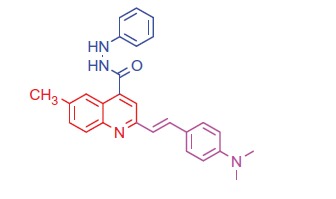


#### 2.1.5. General method for synthesis of compounds 8a,b and 9a,b

To the acid chloride **5** (10 mmol), sodium salt of ethyl 2-cyanoacetate or pentane-2,4-dione [prepared by using sodium ethoxide and ketone derivatives in ethanol (20 ml)] was added and stirred at room temperature overnight. The reaction mixture was filtered, washed with water, dried, and crystallised from DMF.

##### 2.1.5.1. Ethyl 3-(6-bromo-2-(4-(dimethylamino)styryl)quinolin-4-yl)-2-cyano-3-oxopropanoate (8a)

White crystals; yield: 82%; m.p. > 300 °C. ^1 ^H-NMR (400 MHz-DMSO-d_6_): δ 1.22 (t, *J* = 8 Hz, 3 H, CH_3_), 3.14 (s, 6 H, 2CH_3_), 4.20 (q, *J* = 8 Hz, 2 H, CH_2_), 4.60 (s, 1 H, CH-CN), 6.58 (d, *J* = 8 Hz, 1 H, styryl-H), 6.66 (d, *J* = 4 Hz, 1 H, styryl-H), 6.82 (d, 2 H, Ar-H), 7.47 (d, 2 H, Ar-H), 7.52 (d, *J* = 8 Hz, 1 H, Ar-H), 8.00 (s, 1 H, Ar-H), 8.23 (d, *J* = 8 Hz, 1 H, Ar-H), 8.41 (s, 1 H, Ar-H). MS (*m/z*%): 493 (M^+^ + 1, 42.11), 491 (M^+^–1, 34.17), 363 (100). Anal. Calcd for C_25_H_22_BrN_3_O_3_ (492.37): C, 60.99; H, 4.50; N, 8.53. Found: C, 60.95; H, 4.48; N, 8.50.
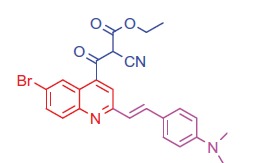


##### 2.1.5.2. Ethyl 2-cyano-3-(2-(4-(dimethylamino)styryl)-6-methylquinolin-4-yl)-3-oxopropanoate (8 b)

White crystals; yield: 80%; m.p. > 300 °C. IR (KBr) γ/cm^−1^: 2210 (C≡N), 1705 (C = O ester), 1628 (C = O ketone). ^1 ^H-NMR (400 MHz-DMSO-d_6_): δ 1.24 (t, *J* = 8 Hz, 3 H, CH_3_), 3.00 (s, 3 H, Ar-CH_3_), 3.10 (s, 6 H, 2CH_3_), 4.18 (q, *J* = 8 Hz, 2 H, CH_2_), 4.53 (s, 1 H, CH-CN), 6.59 (d, *J* = 8 Hz, 1 H, styryl-H), 6.60 (d, *J* = 4 Hz, 1 H, styryl-H), 6.80 (d, 2 H, Ar-H), 7.44 (d, *J* = 8 Hz, 1 H, Ar-H), 7.52 (d, 2 H, Ar-H), 8.05 (s, 1 H, Ar-H), 8.12 (d, *J* = 8 Hz, 1 H, Ar-H), 8.38 (s, 1 H, Ar-H). MS (*m/z*%): 428 (M^+^ + 1, 22.39), 427 (M^+^, 50.11), 299 (100). Anal. Calcd for C_26_H_25_N_3_O_3_ (427.50): C, 73.05; H, 5.89; N, 9.83. Found: C, 73.09; H, 5.92; N, 9.87.
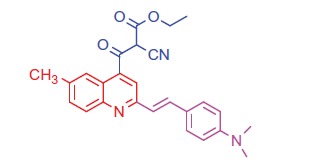


##### 2.1.5.3. 3-(6-Bromo-2-(4-(dimethylamino)styryl)quinoline-4-carbonyl)pentane-2,4-dione (9a)

White crystals; yield: 77%; m.p. > 300 °C. IR (KBr) γ/cm^−1^: 1620 (C = O ketone). ^1 ^H-NMR (400 MHz-DMSO-d_6_): δ 3.00 (s, 6 H, 2CH_3_), 3.37 (s, 6 H, 2COCH_3_), 4.64 (s, 1 H, CH-CO), 6.50 (d, *J* = 8 Hz, 1 H, styryl-H), 6.62 (d, *J* = 4 Hz, 1 H, styryl-H), 6.85 (d, 2 H, Ar-H), 7.49 (d, 2 H, Ar-H), 7.57 (d, *J* = 8 Hz, 1 H, Ar-H), 7.95 (s, 1 H, Ar-H), 8.03 (d, *J* = 8 Hz, 1 H, Ar-H), 8.35 (s, 1 H, Ar-H). ^13^C NMR (100 MHz-DMSO-d_6_): δ 27.2, 41.5, 80.2, 112.4, 114.9, 123.8, 125.0, 126.2, 128.0, 128.2, 129.1, 129.8, 131.0, 132.4, 134.8, 144.5, 154.8, 194.0, 199.7. MS (*m/z*%): 480 (M^+^ + 1, 20.44), 478 (M^+^–1, 31.00), 351 (100). Anal. Calcd for C_25_H_23_BrN_2_O_3_ (479.37): C, 62.64; H, 4.84; N, 5.84. Found: C, 62.60; H, 4.81; N, 5.80.
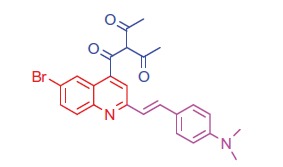


##### 2.1.5.4. 3-(2-(4-(Dimethylamino)styryl)-6-methylquinoline-4-carbonyl)pentane-2,4-dione (9 b)

White crystals; yield: 79%; m.p. > 300 °C. IR (KBr) γ/cm^−1^: 1625 (C = O ketone). ^1 ^H-NMR (400 MHz-DMSO-d_6_): δ 2.93 (s, 6 H, 2CH_3_), 3.11 (s, 3 H, Ar-CH_3_), 3.34 (s, 6 H, 2COCH_3_), 4.60 (s, 1 H, CH-CO), 6.44 (d, *J* = 8 Hz, 1 H, styryl-H), 6.60 (d, *J* = 4 Hz, 1 H, styryl-H), 6.80 (d, 2 H, Ar-H), 7.42 (d, *J* = 8 Hz, 1 H, Ar-H), 7.52 (d, 2 H, Ar-H), 7.92 (d, *J* = 8 Hz, 1 H, Ar-H), 8.04 (s, 1 H, Ar-H), 8.21 (s, 1 H, Ar-H). MS (*m/z*%): 415 (M^+^ + 1, 31.26), 414 (M^+^, 50.37), 286 (100). Anal. Calcd for C_26_H_26_N_2_O_3_ (414.51): C, 75.34; H, 6.32; N, 6.76. Found: C, 75.37; H, 6.37; N, 6.79.
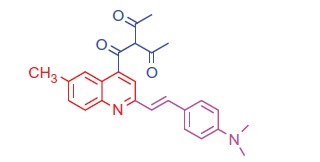


### 2.2. Biological assay

#### 2.2.1. Antitumour activity using MTT assay

The designed compounds were evaluated for their *in vitro* antitumour effect using the standard MTT method against two human tumour cell lines, namely, HepG2 and HCT116^45–48^. The quantitative evaluation of the cytotoxicity was performed using tetrazolium salt MTT (3-(4,5-dimethyl-2-thiazolyl)-2,5-diphenyl-2 *H*-tetrazolium bromide) assay. The cytotoxic activity was expressed as the concentration of the compound that caused 50% growth inhibition (IC_50,_ mean ± SEM) compared with the growth of untreated cells.

#### 2.2.2. EGFR kinase inhibition assay

EGFR kinase activity was determined via enzyme-linked immunosorbent assay (ELISA) in 96-well plates^50^. The EGFR kinase activity for each compound were expressed as IC_50_ values. Data were represented as mean ± SD from three independent experiments, and differences between groups were considered statistically significant at *p* < .05.

### 2.3. Docking methodology

All modelling experiments were conducted with MOE programs running on PC computer (MOE 2008.10, Chemical Computing Group, Inc., Montreal, QC, Canada)^51–54^. Starting coordinates of the X-ray crystal structure of the EGFR enzyme in complex with erlotinib (PDB code 1M17) were obtained from the RCSB Protein Data Bank^29^.

## 3. Results and discussion

### 3.1. Chemistry

**Scheme 1.**
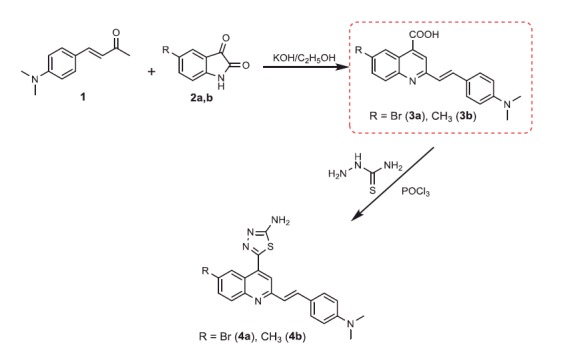
Synthesis of the designed 2-styryl-4-quinoline carboxylic acids, and 1,3,4-thiadiazoles **3a,b** and **4a**,**b**.

**Scheme 2.**
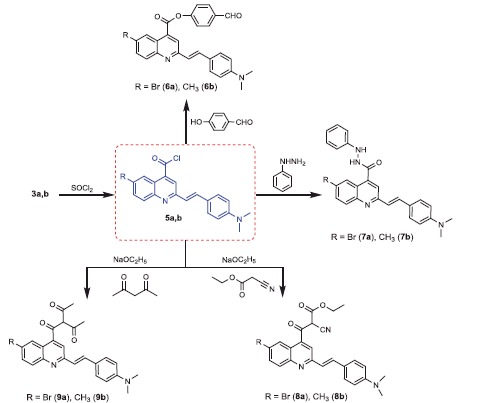
Synthesis of compounds **5a,b–9a**,**b**.

The target compounds were prepared as outlined in Schemes 1 and 2. The structures of the target compounds were established based on elemental analysis, IR, ^1 ^H NMR, ^13^C NMR, and MS data. The starting compounds, 2-(4-(dimethylamino)styryl)-6-substituted quinoline-4-carboxylic acids **3a**,**b**, were prepared through the Pfitzinger reaction, which offers a very convenient synthetic entry to the quinoline-4-carboxylic acid derivatives **3a**,**b** by heating 4–(4-(dimethylamino)phenyl)but-3-en-2-one (**1**) and 5-substituted isatins **2a**,**b** in an aqueous/alcoholic KOH solution (Scheme 1)^55–57^.

The condensation of quinoline-4-carboxylic acids **3a**,**b** with thiosemicarbazide in the presence of phosphorus oxychloride^58^ afforded the 2-amino-1,3,4-thiadiazoles **4a**,**b** (Scheme 1). The IR spectra of the compounds **4a**,**b** showed disappearance of peak at 3400–3448 cm^−1^ (OH group) and peak at 1681 cm^−1^ (C = O group) in compounds **3a**,**b**, while peaks in the ranges 3380–3384 cm^−1^ and 685–690 cm^−1^, attributed to (NH_2_) and (C–S–C) groups, respectively, were observed. In addition, ^1 ^H NMR spectra of compounds **4a**,**b** showed signals in the range of 8.91–8.97 ppm characteristic for the –NH_2_ group with disappearance of –COOH group signal of compounds **3a**,**b** at 11.69 ppm.

Generation of the acid chlorides **5a**,**b** from their corresponding carboxylic acids **3a**,**b** was achieved through heating under reflux with thionyl chloride (Scheme 2). These acid chlorides **5a**,**b** were subjected to reaction with 4-hydroxybenzaldehyde and phenylhydrazine in dimethylformamide containing potassium carbonate to yield 4-formylphenyl 2-(4-(dimethylamino)styryl)-6-substituted quinoline-4-carboxylates **6a**,**b** and 2-(4-(dimethylamino)styryl)-6-substituted N′-phenylquinoline-4-carbohydrazides **7a**,**b**, respectively (Scheme 2). The IR spectra of compounds **6a**,**b** and **7a**,**b** showed absorption bands at 1644–1657 cm^−1^ and 3362–3365 cm^−1^ attributed to formyl and amino groups, respectively. In addition, ^1 ^H NMR spectra of compounds **6a**,**b** showed signals in the range of 9.07–9.13, characteristic of the formyl moieties. The acid chlorides **5a**,**b** were further condensed with either ethyl cyanoacetate or acetyl acetone, to afford compounds **8a**,**b** and **9a**,**b** (Scheme 2). ^1 ^H NMR spectra of compounds **8a**,**b** showed characteristic triplet and quartet signals for the ethyl ester groups at 1.22–1.24 ppm and 4.18–4.20 ppm, respectively. Furthermore, compounds **9a**,**b** showed singlet signals for the methyl ketone groups (O = C–CH_3_) at approximately 3.34–3.37 ppm in addition to a singlet peak at 4.60–4.64 ppm characteristic of the –CH– groups (–CH–CO–CH_3_).

### 3.2. Biological evaluation

#### 3.2.1. Antitumour activity

**Figure 2.**
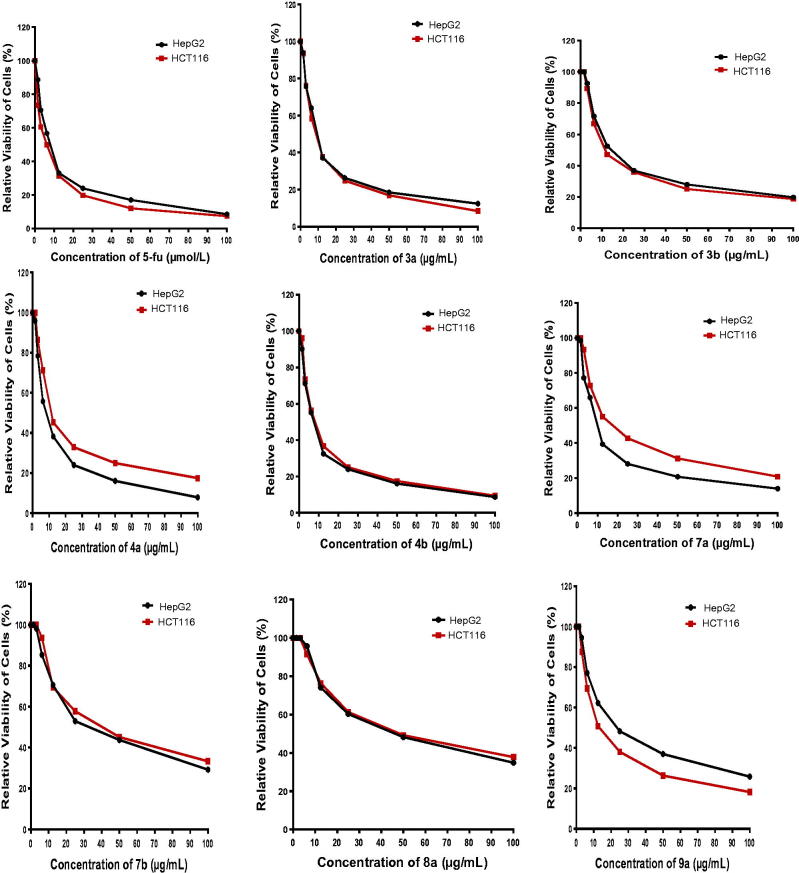
Relative viability of cells (%) against concentration of the newly synthesized compounds.

The antitumour activity of the designed compounds **3a**,**b–9a**,**b** and both reference drugs 5-fluorouracil (5-FU) and afatinib against HepG2 and HCT116 cell lines is shown in Table 1 and Figure 2^45–48^. It is clear that compounds **3a** and **4a**,**b** exhibited the highest antitumour activities against the tested cell lines, with an IC_50_ range of 7.7–14.0 µg/ml, in comparison with the IC_50_ values of the reference drugs 5-FU (IC_50_ range of 5.3–7.9 µg/ml) and afatinib (IC_50_ range of 5.4–11.4 µg/ml). Moreover, 2–(4-(dimethylamino)styryl)-6-methylquinoline-4-carboxylic acid (**3b**) exhibited strong cytotoxic effects against HepG2 and HCT116 cell lines with IC_50_ values of 17.2 and 14.8 µg/ml, respectively. In addition, ethyl 3-(6-bromo-2-(4-(dimethylamino)styryl)quinolin-4-yl)-2-cyano-3-oxopropanoate (**8a**) showed strong activity against the HCT116 cell line (IC_50_ = 16.0 µg/ml) and moderate activity against the HepG2 cell line (IC_50_ = 26.2 µg/ml). On the contrary, compounds **7a** and **8 b** showed moderate efficacy against HepG2 and HCT116 cell lines with an IC_50_ range of 43.7–52.6 µg/ml. Finally, 4-formylphenyl 2-(4-(dimethylamino)styryl)quinoline-4-carboxylates **6a**,**b** and 4-carbonylpentane-2,4-diones 3-(2-(4-(dimethylamino)styryl)quinoline-4-carbonyl)pentane-2,4-diones **9a**,**b** exhibited weak to very weak cytotoxic activity, with an IC_50_ range of 57.3–100 µg/ml.

**Table 1.** *In vitro* antitumour activity of the tested compounds.

Compound no.	*In vitro* cytotoxicity IC_50_ (µg/ml)^a^	
	HepG2	HCT116
**3a**	9.8 ± 0.20	9.0 ± 0.35
**3b**	17.2 ± 1.04	14.8 ± 0.89
**4a**	9.0 ± 0.19	14.2 ± 0.67
**4b**	7.7 ± 0.15	8.8 ± 0.26
**6a**	82.9 ± 4.64	96.6 ± 5.40
**6b**	>100	>100
**7a**	46.1 ± 2.81	49.7 ± 3.00
**7b**	72.8 ± 3.82	61.4 ± 3.76
**8a**	26.2 ± 1.79	16.0 ± 0.88
**8b**	43.7 ± 2.66	52.6 ± 3.92
**9a**	65.4 ± 3.18	57.3 ± 3.07
**9b**	>100	93.1 ± 5.64
5-FU	7.9 ± 0.17	5.3 ± 0.32
Afatinib	5.4 ± 0.25	11.4 ± 1.26

a IC_50_, compound concentration required to inhibit tumour cell proliferation by 50%.

IC_50_, (μg/ml): 1–10 (very strong), 11–25 (strong), 26–50 (moderate), 51–100 (weak), above 100 (non-cytotoxic).

#### 3.2.2. EGFR inhibitory activity

The mechanism of antitumour activity of the target compounds was studied using ELISA-based EGFR-TK assay^50^. Five compounds with the highest antitumour activities were evaluated against EGFR kinase activity assays with sorafenib and erlotinib as the reference drugs. IC_50_ values of the tested compounds were calculated and are listed in Table 2, where compounds **3a** (IC_50_ = 2.23 µM) and **4b** (IC_50_ = 1.11 µM) showed the highest inhibitory activity against EGFR, compared to the other tested compounds. The activities of compounds **3b** (IC_50_ = 8.01 µM), **4a** (IC_50_ = 8.78 µM), and **8a** (IC_50_ = 16.01 µM) against EGFR were found to be weakly active comparable to those of sorafenib (IC_50_ = 1.14 µM) and erlotinib (IC_50_ = 0.1 µM). Based on these results, we can conclude that EGFR-TK inhibitory activity of the target compounds is correlated to their antitumour activities against HepG2 and HCT116.

**Table 2.** IC_50_ values of the designed compounds toward EGFR kinase and docking interaction energy.

Compound no.	Enzymatic. IC_50_ (μM)^a^	Docking interaction energy (kcal/mol)
**3a**	2.23	–18.54
**3b**	8.01	–
**4a**	8.78	–
**4b**	1.11	–20.89
**8a**	16.01	–
Sorafenib	1.14	–
Erlotinib	0.10	–29.01

a Data represent mean ± SD, *n* = 3, *p < .05.

### 3.3. Molecular docking study

The inhibitory activities of compounds **3a** and **4b** on EGFR kinase prompted us to carry out molecular docking into the putative binding site of EGFR kinase. Both compounds **3a** and **4b** were flexibly docked into the active site of EGFR kinase along with the reference inhibitor erlotinib (PDB code: 1M17)^29^. All docking calculations were performed using MOE 2008.10 software^7b^^,^^51–54^_._

**Figure 3.**
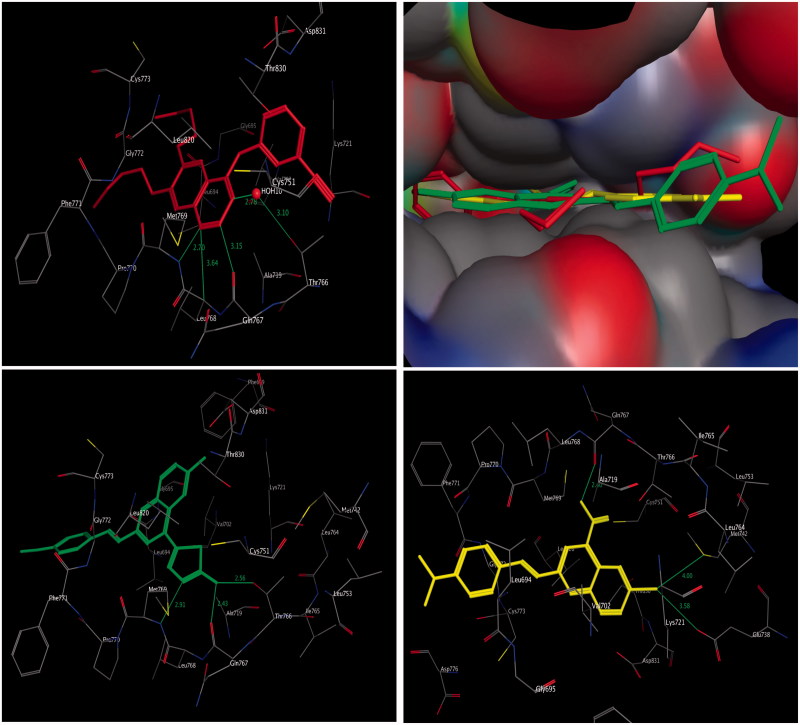
Three-dimensional interactions of erlotinib (upper left panel), compounds **4b** (lower left panel), and **3a** (lower right panel) with the receptor pocket of EGFR kinase. Hydrogen bonds are shown as green line. Upper right panel shows superimposition of compounds **4b** (green coloured) and **3a** (yellow coloured) on erlotinib (red coloured) inside the pockets of the active site.

The interaction energies of compounds **3a** and **4b** and erlotinib, docked into the active site of EGFR, were –18.54, –20.89, and –29.01 kcal/mol, respectively (Table 2 and Figure 3). The molecular docking results of the most active compound **4b** demonstrated a hydrophobic interaction of the quinoline ring with surrounding amino acids, such as Val^702^, Leu^694^, and Leu^820^. The substituent group at C-4 of the quinoline ring is the main moiety affecting the binding mode of compound **4b** in both activation and catalytic loops, where a 2-aminothiadiazole ring uniquely formed trifurcated hydrogen bonds with the distinctive residue Met^769^, Gln^767^, and Thr^766^. Moreover, the 2-styryl fragment of compound **4b** was firmly extended to the backbone, similar to the 6,7-dialkoxy moiety of erlotinib, augmenting the recognition and the overall inhibitory activity (Figure 3, lower left panel).

Compound **3a** binds in a similar manner to compound **4b**, where hydrophobic interaction is clearly observed among amino acid residues Val^702^, Leu^694^, and Leu^820^ and quinoline core. It was found that the carboxylic group at position-4 of the quinoline core was clearly recognised, with hydrogen bonding to the amino acid residue Gln^767^, while this carboxylic group was shifted away from the distinctive amino acid residue Met^769^. Moreover, the bromine atom at position-6 formed bifurcated hydrogen bonds with the amino acid residue Met^742^ and Glu^738^ (Figure 3, lower right panel). It is clear that the results of the molecular docking can be used to design novel quinoline derivatives with potential antitumour activity and binding to EGFR kinase.

## 4. Conclusions

Novel 4,6-disubstituted 2-SQ derivatives **3a**,**b**–**9a**,**b** have been synthesized, and their antitumour activity and EGFR inhibition have been evaluated. Among the tested compounds, **3a** and **4a**,**b** (IC_50_ ≅ 7.7–9.8 µg/ml) were identified as the most potent antitumour agents against HepG2 and HCT116 cancer cell lines, with activity comparable to that of 5-FU (IC_50_ ≅ 5.37–7.9 µg/ml) and afatinib (IC_50_ ≅ 5.4 –11.4 µg/ml). Moreover, compound **3b** exhibited strong antitumour activities against HepG2 and HCT116 cancer cell lines with IC_50_ values of 17.2 and 14.8 µg/ml, respectively. Compounds **3a** and **4b** have moderate inhibitory activity on EGFR with IC_50_ values of 2.23 and 1.22 µM, respectively. Accordingly, both compounds **3a** and **4b** are expected to exert their antitumour activity through inhibition of EGFR. A molecular docking study was conducted for compounds **3a** and **4b** and the putative binding site of EGFR kinase, which revealed a binding mode similar to that of the reference inhibitor erlotinib.
